# Transcriptome data from three endemic Myrtaceae species from New Caledonia displaying contrasting responses to myrtle rust (*Austropuccinia psidii*)

**DOI:** 10.1016/j.dib.2018.12.080

**Published:** 2019-01-03

**Authors:** Julia Soewarto, Chantal Hamelin, Stéphanie Bocs, Pierre Mournet, Hélène Vignes, Angélique Berger, Alix Armero, Guillaume Martin, Alexis Dereeper, Gautier Sarah, Fabian Carriconde, Laurent Maggia

**Affiliations:** a Institut Agronomique néo-Calédonien (IAC), 98848 Nouméa, New Caledonia; b CIRAD, UMR AGAP, F-34398 Montpellier, France; c IRD, UMR IPME (IRD-UM2-Cirad) 911, avenue Agropolis, BP 64501, 34394 Montpellier Cedex 5, France; d South Green Bioinformatics Platform, Montpellier, France; e CIRAD, UMR AGAP, 98800 Nouméa, New Caledonia; f AGAP, Univ Montpellier, CIRAD, INRA, Montpellier SupAgro, Montpellier, France

## Abstract

The myrtle rust disease, caused by the fungus *Austropuccinia psidii*, infects a wide range of host species within the Myrtaceae family worldwide. Since its first report in 2013 in New Caledonia, it was found on various types of native environments where Myrtaceae are the dominant or codominant species, as well as in several commercial nurseries. It is now considered as a significant threat to ecosystems biodiversity and Myrtaceae-related economy. The use of predictive molecular markers for resistance against myrtle rust is currently the most cost-effective and ecological approach to control the disease. Such an approach for neo Caledonian endemic Myrtaceae species was not possible because of the lack of genomic resources. The recent advancement in new generation sequencing technologies accompanied with relevant bioinformatics tools now provide new research opportunity for work in non-model organism at the transcriptomic level.

The present study focuses on transcriptome analysis on three Myrtaceae species endemic to New Caledonia (*Arillastrum gummiferum*, S*yzygium longifolium* and T*ristaniopsis glauca*) that display contrasting responses to the pathogen (non-infected vs infected). Differential gene expression (DGE) and variant calling analysis were conducted on each species. We combined a dual approach by using 1) the annotated reference genome of a related Myrtaceae species (*Eucalyptus grandis*) and 2) a de novo transcriptomes of each species.

**Specifications table**

**Table:** 

Subject area	Genetics and Transcriptomics
More specific subject area	Transcriptomics of Myrtaceae species
Type of data	Table, figure
How data was acquired	Leaves of individual plant from three endemic Myrtaceae species from New Caledonia were sampled for total RNA extraction. Paired-end library were prepared and RNA-Sequencing was performed by the Illumina HiSeq™ 2500 system. The obtained data was subjected to 1) *de novo* transcriptome assembly, 2) alignment to a reference genome and de novo transcriptome assembly, 3) differential gene expression and 4) variant calling analysis.
Data format	*Raw data FASTQ file, , analyzed*
Experimental factors	Non-infected and infected individuals from *Arillastrum gummiferum, Syzygium longifolium, Tristaniopsis glauca* exposed to myrtle rust *(Austropuccinia psidii).*
Experimental features	For the RNA-Sequencing and transcriptome analysis, a total of 24 leaves samples from three host species have been collected: three infected and three non-infected individuals from *A. gummiferum*, two infected and five non-infected individuals from *S. longifolium*, four infected and one non-infected individuals from *T. glauca* from the nursery, four infected and two non-infected individuals from *T. glauca* from a natural population in a protected reserve.
Data source location	The nursery was located in Farino, South Province, New Caledonia (Long 165.772024:, Lat: -21.663800). The protected reserve was located in Bois du Sud, South Province, New Caledonia (Long 166.758640:, Lat: -22.169974)
Data accessibility	All raw data for *Arillastrum gummiferum*, *Syzygium longifolium* and *Tristaniopsis glauca* and the processed data (*de novo* transcriptome assemblies, transcriptome annotations and differential gene expression files) obtained in this study were deposited in the National Center for Biotechnology Information (NCBI) Gene Expression Omnibus (GEO) with the Superseries accession number GSE106750 and the subseries accession numbers GSE106735, GSE106736, GSE106738, GSE106740, GSE106741, GSE106746, GSE106747 and GSE106749.
	The Superseries is available at https://www.ncbi.nlm.nih.gov/geo/query/acc.cgi?acc=GSE106750
	The VCF files can be downloaded at http://myrtaceae-omics.southgreen.fr/node/8 provided you are logged and have accepted the Terms of Use of these data.
Related research article	Soewarto J, Carriconde F, Hugot N, Bocs S, Hamelin C, Maggia L. Impact of *Austropuccinia psidii* in New Caledonia, a biodiversity hotspot. Forest
	Pathology. 2018;48 (2). doi:10.1111/efp.12402.

**Value of the data**

• These are the first *de novo* transcriptomes of *Arillastrum gummiferum*, *Syzygium longifolium* and *Tristaniopsis glauca.*
• The obtained transcriptomes data will be useful for further studies of the evolution of Myrtaceae or comparative genomics.
• The data (*de novo* transcriptomes, reads assemblies, differential gene expression, SNP calling, etc…) will provide new valuable genetic resources for investigations of myrtle rust interactions and resistance-related pathways within Myrtaceae plant family.

## 1 Data

The transcriptomes were extracted from leaf samples of individuals of *Myrtaceae* species endemic to New Caledonia (*Arillastrum gummiferum*, *Syzygium longifolium* and *Tristaniopsis glauca*) naturally infected by the fungal pathogen *Austropuccinia psidii* (myrtle rust) and displaying contrasting responses to the infection. Due to facilities restrictions regarding the artificial inoculation of the pathogen in New Caledonia, the natural infection method has been chosen to rapidly screen the infection status of these species. A previous study based on natural myrtle rust infection concluded that *A. gummiferum*, *S. longifolium* and *T. glauca* were all susceptible to the disease and displayed variations in the disease incidence and severity [1].

A total of 24 leaves samples from the three species have been collected for the RNA-Sequencing on: three infected and three non-infected individuals from *A. gummiferum*, two infected and five non-infected individuals from *S. longifolium*, four infected and one non-infected individuals from *T. glauca* from a commercial nursery located in Farino (FAR), four infected and two non-infected individuals from *T. glauca* from the protected reserve of Bois du Sud (BDS). After sequencing, 48 RNA-Seq fastq files (paired end Illumina HiSeq) have been generated.

A total of four *de novo* transcriptomes have been implemented in this study. The individual displaying the most reads at sequencing was chosen as a representing of each species (*A. gummiferum*, *S. longifolium*, *T. glauca*) and location (FAR, BDS).

Height differential gene expression analysis (DGEA) were conducted: four by using each species’s reads mapped on the annotated reference genome of *Eucalyptus grandis* and four by using the reads mapped on each species corresponding *de novo* transcriptome assembly.

Sixteen variant calling analysis where conducted for the four group of species using two types of reference sequences for the mapping (*E. grandis* genome and *de novo* transcriptome assemblies) and two types of variant calling methods (in-house SNP calling and GATK methods).

## 2 Experimental design, materials and methods

### 2.1 Plant material

Individuals from the three following species *Arillastrum gummiferum*, *Syzygium longifolium* and *Tristaniopsis glauca*, were sampled from a commercial nursery located in Farino, New Caledonia. The infection status of each individuals has been monitored at least one month before the sampling to ensure that the natural infection by myrtle rust was effective. Leaf samples from individual plants of *T. glauca* have also been sampled from a natural population occurring in the protected reserve of Bois Du Sud, New Caledonia. To distinguish the individual plants from the two locations, the natural population from Bois Du Sud will be referred as *T. glauca* BDS and the plants originated from the nursery will be referred as *T. glauca* FAR. Thus, this study counts four group of species and population as followed: *A. gummiferum*, *S. longifolium*, *T. glauca* FAR and *T. glauca* BDS.

When *A. psidii* successfully infects a host, disease symptoms can appear within 12 days and are visually characterized by the formation of pustules covered by yellow and powdery urediniospores [2]. The disease can infect various plants parts including actively growing leaves, shoots, fruits, flowers and buds [2], [3]. Although natural infection was showed to be effective to infect at least once every host plants standing in the nursery during the previous monitoring [1], it does not constitute a reliable way to conclude on the resistant status of the individual plants that did not display any sign of symptoms. Thus, the present study will consider that all the individuals that showed signs of myrtle rust symptoms at the sampling time or during a previous monitoring will be classed as infected; and all the individuals that never showed sign of myrtle rust symptoms will be classed as non-infected.

**Fig. 1:**
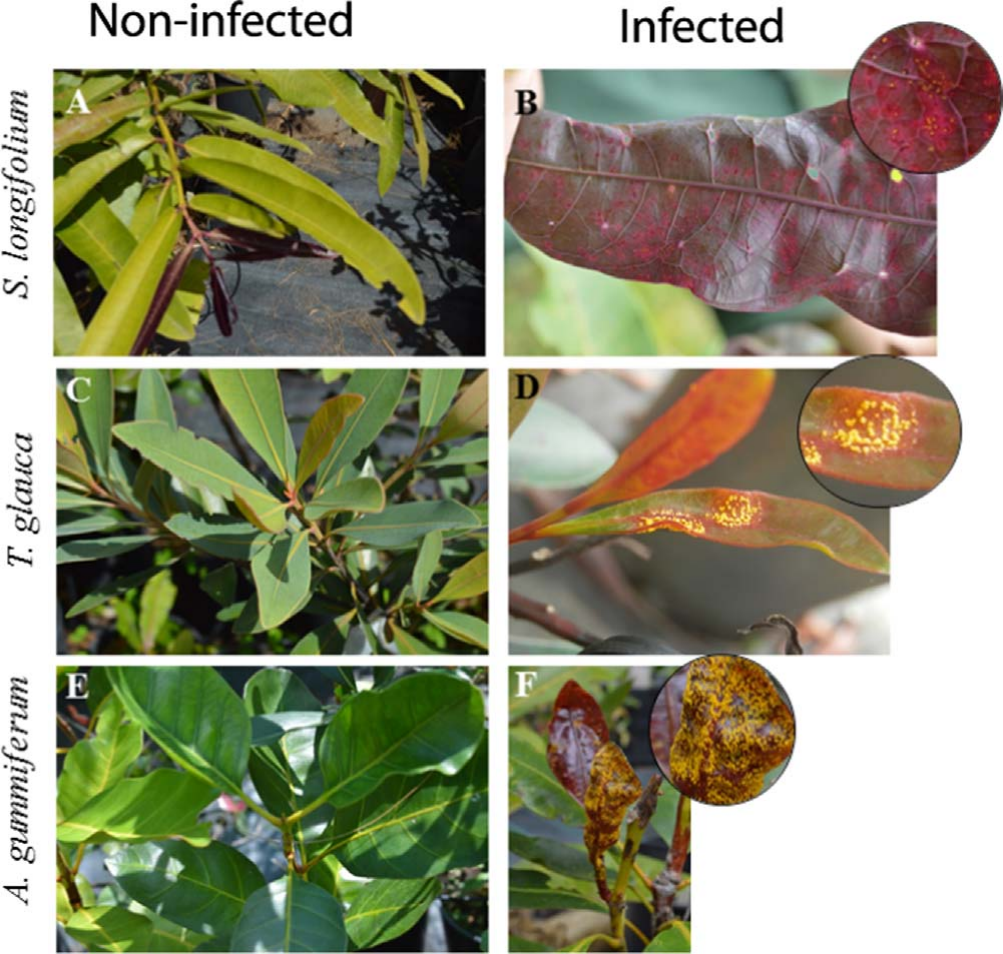
Illustration of the three Myrtaceae species in this study. For each species: Left pictures show non-infected individual and right ones show myrtle rust symptoms on infected individual.

All the samples have been harvested at the same time, in April the 28th, 2015 for the plants in the nursery and in May the 1st, 2015 for the plants in the protected reserve of Bois Du Sud. Each time the most recent leaves were always chosen for harvesting (Fig. 1). All samples were frozen at the time of collection and then stored at −80 °C until total RNA was extracted. The sampling details are provided in Table 1.

**Table 1:** Detailed sampling of three Myrtaceae species for RNA-seq analysis.

**Sample name**	**ID sampling**	**Biological material**	**Organism**	**Sampling site**	**Phenotype toward myrtle rust infection**
Sample 1	Ag19	leaf	*Arillastrum gummiferum*	nursery	infected
Sample 2	Ag28	leaf	*Arillastrum gummiferum*	nursery	infected
Sample 3	Ag2	leaf	*Arillastrum gummiferum*	nursery	non-infected
Sample 4	Ag3	leaf	*Arillastrum gummiferum*	nursery	non-infected
Sample 5	Ag4	leaf	*Arillastrum gummiferum*	nursery	non-infected
Sample 6	Ag6	leaf	*Arillastrum gummiferum*	nursery	infected
Sample 7	Syl10	leaf	*Syzygium longifolium*	nursery	non-infected
Sample 8	Syl13	leaf	*Syzygium longifolium*	nursery	infected
Sample 9	Syl15	leaf	*Syzygium longifolium*	nursery	infected
Sample 10	Syl18	leaf	*Syzygium longifolium*	nursery	non-infected
Sample 11	Syl2	leaf	*Syzygium longifolium*	nursery	non-infected
Sample 12	Syl4	leaf	*Syzygium longifolium*	nursery	non-infected
Sample 13	Syl7	leaf	*Syzygium longifolium*	nursery	non-infected
Sample 14	Tg2	leaf	*Tristaniopsis glauca*	nursery	infected
Sample 15	Tg3	leaf	*Tristaniopsis glauca*	nursery	non-infected
Sample 16	Tg4	leaf	*Tristaniopsis glauca*	nursery	infected
Sample 17	Tg5	leaf	*Tristaniopsis glauca*	nursery	infected
Sample 18	Tg6	leaf	*Tristaniopsis glauca*	nursery	infected
Sample 19	V1	leaf	*Tristaniopsis glauca*	natural field	infected
Sample 20	V2	leaf	*Tristaniopsis glauca*	natural field	infected
Sample 21	V3	leaf	*Tristaniopsis glauca*	natural field	non-infected
Sample 22	V4	leaf	*Tristaniopsis glauca*	natural field	non-infected
Sample 23	V6	leaf	*Tristaniopsis glauca*	natural field	infected
Sample 24	V7	leaf	Tristaniopsis glauca	natural field	infected

### 2.2 Total RNA extraction

Total RNA was extracted using 3–10 g of fresh material from the cetyltrimethylammonium bromide-based protocol (CTAB) [4]. Briefly, 3–10 g of frozen leaves from each individual was ground in liquid nitrogen using a mortar and pestle. Then 3–10 mL of pre-heated extraction buffer (2% CTAB, 2% polyvinylpyrrolidone, PVP-40 (2% w/v), 2% β-mercaptoethanol, 100 mM Tris–HCl, 25 mM EDTA and 2 M NaCl) was added to the ground samples, and incubated at 65 °C for 30 min. An equal volume of mixture of chloroform:isoamyl alcohol (24:1) was added and mixed immediately for 2 min using a vortex mixer. The samples were then centrifuged at 10,000*g* for 10 min. The upper aqueous phase was transferred to new tubes and 1/3 volume of 10 M LiCl was added. The samples were mixed and stored at 4 °C overnight. The samples are then centrifuged at 18,000*g* for 20 min. The supernatant was removed and the pellet is washed with 75% ethanol and air-dried. The pellet was suspended in 30 μl of RNase-free water and 70 μl of SSTE buffer (1M NaCl, SDS (0.5% w/v), 10 mM Tris–HCl, 1 mM EDTA). An equal volume of acid:phenol:chloroform:isoamyl alcohol (25:24:1) is added to each sample, and vortexed. The samples were then centrifuged at 12,000 g for 10 min and the upper aqueous phase was transferred to new tubes with 2 volumes of cooled ethanol 100% and 1/10 volumes of NaAc (pH5.2). The samples are then mixed and incubated at −20 °C for 2 h. The samples were centrifuged at 18,000*g* for 20 min. The supernatant was removed and the pellet was washed three times with 75% ethanol before being air-dried. Finally, the pellet is resuspended in 30 μl of RNase-free water. The RNA extraction is followed by removal of DNA with the TURBO DNA-*free*^™^ kit (Ambion) according to the manufacturer’s instructions. RNA quantity and quality control was performed using a 2100 Bioanalyzer (Agilent Technologies).

### 2.3 cDNA library preparation and sequencing

Paired-end Illumina mRNA libraries were generated using the TruSeq RNA-Seq Sample Prep kit according to the manufacturer’s protocol (Illumina Inc., San Diego, CA, USA). Briefly, Poly-A containing mRNA molecules were isolated using poly-T oligo-attached magnetic beads. The purified mRNA was then chemically fragmented. Reverse transcription of first- and second-strand of cDNA are performed and were followed by end repair. Single ‘A’ nucleotide is added to the 3′ ends of each fragment before ligation of adapters. The purified cDNA templates were enriched by PCR to form libraries of 300 pb. Each indexed cDNA library was verified and quantified using a 2100 Bioanalyzer (Agilent Technologies). The final libraries were then quantified by qPCR with the KAPA Library Quantification Kit for Illumina Sequencing Platforms (Kapa Biosystems Ltd, SA) and normalized to 20 nM before being pooled. The quality of our samples was suitable for the Illumina HISAT 2500 requirement sequencing (RIN between 6.9 and 8.4). Details of RNA samples were supplied in Supplementary table 1. Sequencing was then conducted on a single lane of a flow cell on Illumina HiSeq^™^ 2500 (Genotoul platform, INRA) as paired-ends reads of length 150 bp.

### 2.4 RNA-seq data processing

**Fig. 2:**
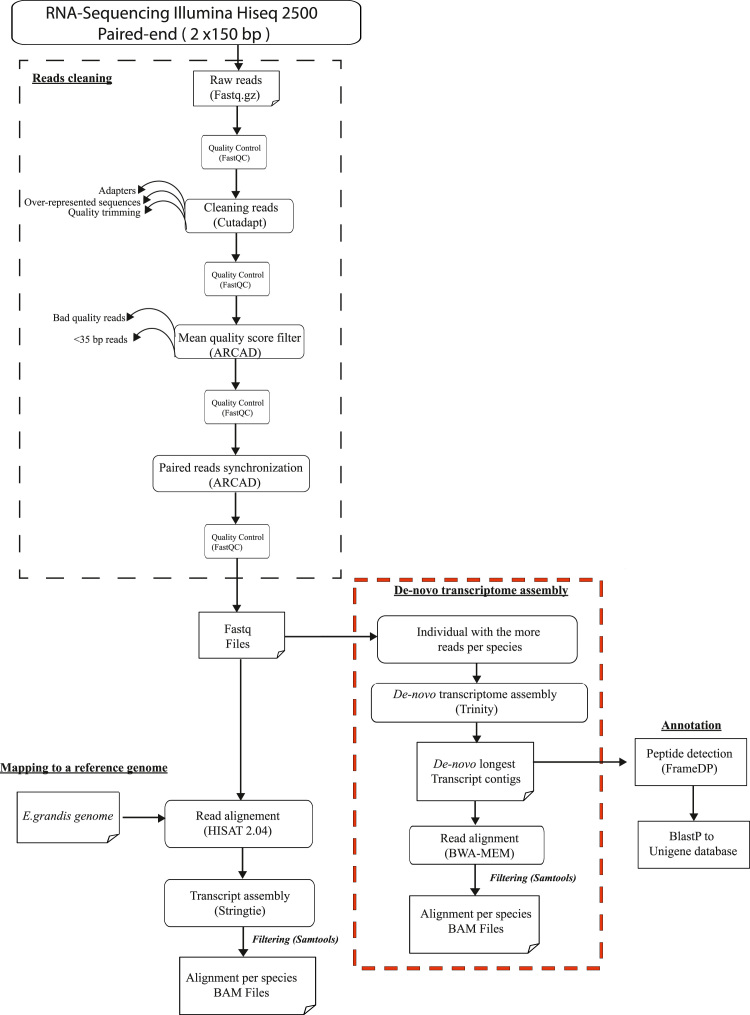
Bioinformatics pipeline showing the different steps involved in RNA-seq analysis until alignment to the two kind of reference.

RNA-seq data cleaning processing was followed according the ARCAD (Agropolis Resource Center for Crop Conservation, Adaptation and Diversity) workflow analysis (http://arcad-bioinformatics.southgreen.fr/) in command line as described in Fig. 2. Scripts are available on the SouthGreen GitHub repository (https://github.com/SouthGreenPlatform/arcad-hts).

#### 2.4.1 Raw reads cleaning

**Fig. 3:**
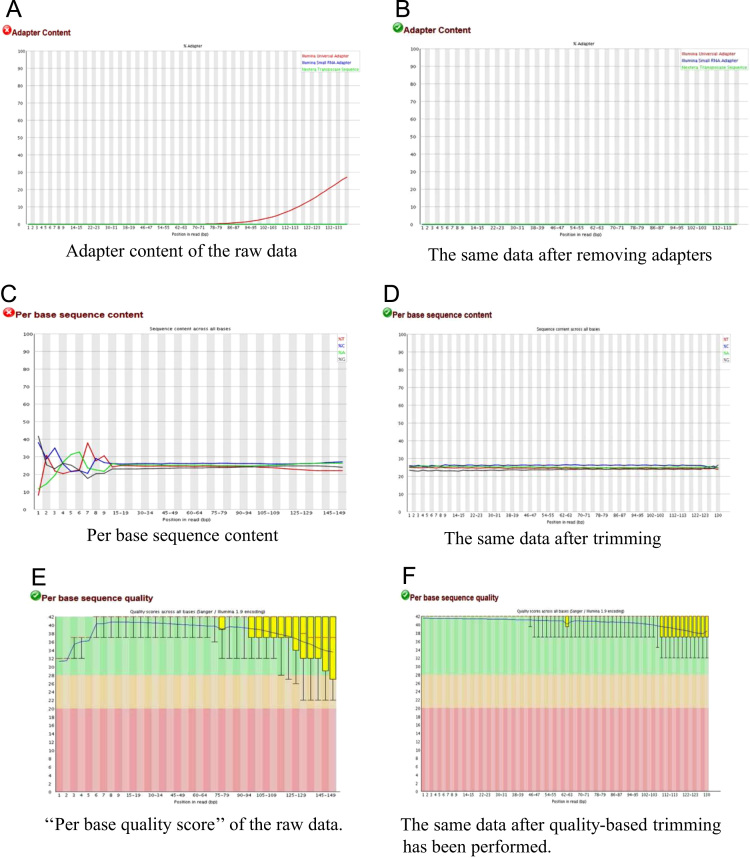
Quality control statistics generated by FastQC for individual Syl18 (*S. longifolium*) at different stages of the data cleaning process.

RNA-sequencing for the three species, produced a total of 668,165,595 raw reads. The quality of paired-end raw reads in fastq format was assessed using FastQC sofware (http://www.bioinformatics.babraham.ac.uk/projects/fastqc/) with the script arcad_hts_0_fastqc_in_chains.pl. Raw reads were then processed with Cutadapt [5] using the script arcad_hts_1_cutadapt_in_chain.pl and the TruSeq index sequences corresponding to the samples. We also used Cutadapt to improve mean reads quality by trimming the start and the end of each reads. We then filtered the reads on the basis of their mean quality score, keeping those with a mean quality higher than 30 using arcad_hts_2_Filter_Fastq_On_Mean_Quality.pl. Reads with length inferior to 35 bp were discarded. Thereafter, single reads (i.e. those for which the mate pair was discarded in the previous steps) were separated from paired-reads using arcad_hts_3_synchronized_paired_fastq_end.pl. Each processing steps for reads cleaning were followed by a FastQC quality control of reads (arcad_hts_0_fastqc_in_chains.pl). Each pre-processing cleaning steps is illustrated in Fig. 2. After the cleaning stage, we kept 96% of the initial reads (644,707,222 reads). An example of the efficiency of quality control on our data is shown in Fig. 3. A summary of the RNA-seq raw and clean data is presented in Table 2 and detailed in Supplementary table 2.

**Table 2:** Number of raw and cleaned reads from the three species.

	*A. gummiferum*	*S. longifolium*	*T. glauca*-FAR	*T. glauca*-BDS
Number of libraries/individuals	6	7	5	6
Length of raw reads (bp)	150	150	150	150
Total number of raw reads	176,074,893	200,293,564	137,602,172	154,194,966
Total number of clean reads	169,892,296	193,481,676	132,410,558	148,922,692
Length of clean reads (bp)	130	130	130	130

#### 2.4.2 Reference-guided method

##### 2.4.2.1 Aligning to a reference genome from a related species

The reference genome assembly and annotation of *E. grandis* were downloaded from NCBI RefSeq portal (https://www.ncbi.nlm.nih.gov/assembly/GCF_000612305.1/). FASTQ files containing cleaned reads for each individual were mapped to reference genome *Eucalyptus grandis* (EUCGR) using HISAT 2.04 (hierarchical indexing for spliced alignment of transcripts) [6]. The aligned data were passed to StringTie [7] for transcript assembly. A reference genome annotation file in GFF3 format (intron/exon positions) was provided to guide the transcripts assembly. The resulting binary files (.bam) were then filtered on quality using SAMtools view (with parameters -q 0 -F 4) to remove unmapped and multimapped reads [8]. Finally, the reads were sorted using SAMtools sort. Mapping statistics were verified using SAMtools flagstat [8] (Table 3). We obtained mapping rates of approximately 68% for *A. gummiferum*, *S. longigolium*, *T. glauca*- FAR and *T. glauca*-BDS. And around 5% of the total reads mapped correspond to singletons (Table 4).

**Table 3:** Alignment statistics indicative of reads aligned to the assembled transcriptome using SamTools flagstat.

Species	Sample name	***E. grandis*****ref. genome**			***De novo*****transcriptome**		
		Total reads mapped	Properly paired (%)^a^	Singletons mapped (%)^b^	Total reads mapped	Properly paired (%)^a^	Singletons mapped (%)^b^
*A. gummiferum*	Ag19	22,195,213	70	4.8	19,953,353	95	0.1
	Ag28	23,611,351	71	4.8	20,537,263	96	0.1
	Ag2	25,655,914	68	4.5	23,462,050	96	0.1
	Ag3	27,318,150	68	4.6	24,139,896	96	0.1
	Ag4	18,576,164	69	4.5	17,399,264	97	0.1
	Ag6	21,277,541	74	4.2	17,259,913	96	0.1
*S. longifolium*	Syl10	26,685,822	68	6.1	25,218,899	96	0.1
	Syl13	16,676,981	65	5.0	16,426,865	97	0.1
	Syl15	17,382,267	63	5.2	15,522,277	97	0.1
	Syl18	19,263,137	69	5.4	18,980,838	96	0.1
	Syl2	23,370,932	67	5.3	22,382,429	96	0.1
	Syl4	23,806,097	66	5.7	21,148,273	95	0.1
	Syl7	19,456,046	66	5.1	19,209,000	97	0.1
*T. glauca-*FAR	Tg2	18,287,633	71	5.5	16,225,569	96	0.2
	Tg3	20,985,018	70	5.5	19,345,206	97	0.2
	Tg4	19,284,476	70	6.9	19,094,992	95	0.3
	Tg5	19,370,817	65	4.2	17,549,513	97	0.1
*T. glauca-*BDS	Tg6	17,264,359	67	4.9	16,126,662	96	0.1
	V1	12,259,586	58	3.6	12,446,883	99	0.0
	V2	20,957,190	70	4.6	18,089,903	98	0.1
	V3	18,561,244	66	4.1	17,126,183	98	0.1
	V4	17,177,833	66	5.1	17,252,577	96	0.1
	V6	22,656,615	66	4.9	20,231,181	97	0.0
	V7	19,387,135	70	5.7	17,984,129	97	0.1

a Number of proper pairs in proportion to the total reads mapped

b Number reads where one from a pair in proportion to the total mapped

**Table 4:** Overlapped and uniques SNPs called using two different calling methods (GATK and in-house script) from mapping using *E. grandis* reference genome.

**Species**	**Methods**	**filtered SNP counts**	**% unique SNP positions**	**% shared SNP positions between GATK and inhouse script methods**
*A. gummiferum*	GATK (Haplotype Caller)	142,294	66	34
	Inhouse script	73,765	34	66
*S. longifolium*	GATK (Haplotype Caller)	181,967	79	21
	Inhouse script	68,106	45	55
*T. glauca*-BDS	GATK (Haplotype Caller)	148,484	75	25
	Inhouse script	67,115	44	56
*T. glauca*-FAR	GATK (Haplotype Caller)	137,073	60	40
	Inhouse script	83,243	34	66

##### 2.4.2.2 Calling SNPs using *E. grandis* reference genome

**Fig. 4:**
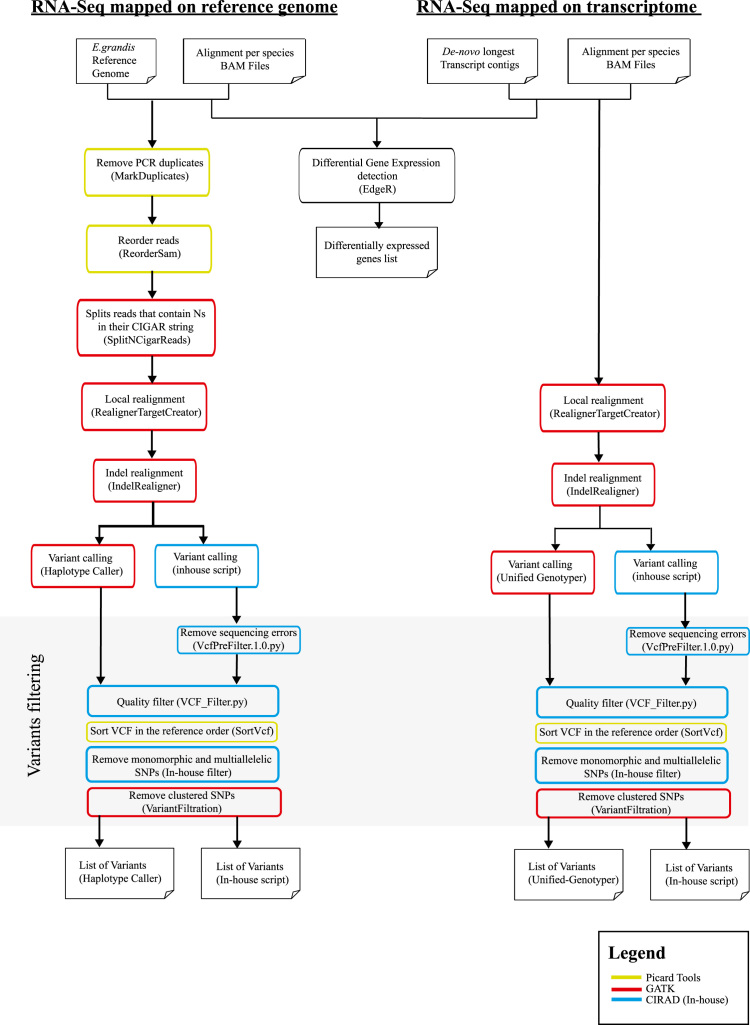
Analytic pipeline for differential gene expression (DGE) and Variant calling (SNP).

Variant calling algorithms compare mapped reads to a reference genome and identify potential variants. The analysis pipeline we used is illustrated in Fig. 4. We followed the best practices recommended by the GATK (Genome Analysis Toolkit) pipeline [9]. Consistent with GATK’s recommendations, mapped reads against *E. grandis* reference genome have been submitted to cleaning process before SNP calling step. We used Picard tools to remove PCR duplicates (MarkDuplicates) and reorder reads to match the contigs according to the reference genome (ReorderSam). Then, we used the GATK tool SplitNCigarReads (with parameters -RMQF 255 -RMQT 60 -U ALLOW_N_CIGAR_READS), which splits reads into exon segment and hard-clip any sequences overhanging into the intronic regions. The reads were realigned around INDELS and base quality values were recalibrated using RealignerTargetCreator and IndelRealigner GATK tools. Once the reads were pre-processed with Picard and GATK tools, variant calling was undertaken by two programs: Haplotype Caller (GATK) and an in-house script (Martin et al., *in prep.*, Baurens et al., *in prep.*). Contrary to GATK, which uses statistics based on population genetics (which is not the case here), the in-house program only count the number of reads supporting each bases (A, T, G, C) at each covered sites for each accession. Based on this base count for each accession, a genotype was emitted based on a binomial test. We obtained between 3 and 5 million of SNPs with Haplotype Caller for each species, and the in-house script identified between 8 and 11 million of SNPs (Supplementary Table 3).

##### 2.4.2.3 Variant filtering mapping to *E. grandis* genome

Several filtering criteria were used to exclude the less reliable SNPs from all the raw called variants dataset (Fig. 4). Because in-house SNP calling method implied to detect any difference between the reads and the reference (including false positive SNP), the initial outputs were too large and must be pre-filtered in order to allow comparison with those resulting from GATK SNP calling method. Firstly we applied an in-house pre-filter script (VcfPreFilter.1.0.py) on SNPs resulting from the in-house SNP calling method in order to remove SNPs due to sequencing errors. This pre-filter included the following parameters: a minimum site coverage (5 reads), a maximal site coverage (1000 reads) per SNP, a minimum allele frequency (0.01) and a minimum allele coverage (2 reads per allele). Then for all datasets (from GATK and in-house SNP calling methods) we used an in-house VCF filter script (VCF_Filter.py) to keep only the more robust SNPs according to the following criteria: a minimum coverage (10), a maximal coverage (1000), a minimum frequency (0.05), a minimum allele coverage (3) to keep a genotype; and no missing data were allowed to keep a variant site. Another in-house script was then used to remove monomorphic and multiallelic variants. And finally, as recommended by GATK, we used the Variant Filtration program to remove clustered SNP: in a window size of 10 pb we considered that 3 SNPS constitute a cluster. The final SNP set comprised the identified SNPs that had passed all filters. We compared the overlap among SNP positions obtained for each calling methods using vcf-compare from VCF tools [10] (Table 4 ).

With *E. grandis* genome as reference for mapping, we found around 30% of shared SNPs positions using Haplotype Caller, and around 60% of shared SNPs positions using In-house script (Table 4).

### 2.5 *De novo* method

#### 2.5.1 *De novo* transcriptome assembly

We chose the individual with the highest read number after cleaning for each species for the *de novo* assembly as described by Sarah et al. [11]. *De novo* transcriptome assembly was carried out using Trinity software with 50 GB of memory (Inchworm, Chrysalis, and Butterfly modules) [12]. Briefly, overlapping k-mers are extracted from the cleaned paired-reads. Inchworm module assembled the reads into contigs. Next, Chrysalis module ranked Inchworm contigs into clusters and constructed complete de Bruijn graphs for each cluster. Finally, Butterfly module processed the individual graphs in parallel to reconstruct transcript sequences in a manner that reflects the original cDNA molecules. To avoid redundant transcripts, we kept the longest isoform for each “trinity gene”. Assembly statistics (N50, contig length, GC content, etc) were computed by TrinityStats.pl embedded in Trinity (Table 5; Supplementary table 2). Transcriptome assembly for *A. gummiferum* resulted in 117 839 putative transcripts with an average contig length of 501 bp and N_50_ of 1378 base pairs. Transcriptome assembly for *S. longifolium* yielded a total number of 89,782 putative transcripts with an average contig length of 530 bp in length and N_50_ of 1406 base pairs. Transcriptome assembly for *T. glauca-FAR* resulted in 108,823 putative transcripts, with an average contig length of 547 bp and N_50_ of 1396 base pairs. And finally, *de novo* transcriptome assembly for *T. glauca-BDS* resulted in 74,684 putative transcripts with an average contig length of 525 bp in length and N_50_ of 1315 base pairs. However, Trinity *de novo* assembly resulted in larger number of transcripts than expected number of genes, likely because of alternative splicing. To avoid redundant transcripts, we kept the longest isoform for each “gene” identified by TRINITY (unigene). Overall size of filtered *de novo* assembly yielded 57 Mb for *A. gummiferum*, 47 Mb for *S. longifolium*, 58 Mb for *T. glauca*-FAR and 39 Mb for *T. glauca*-BDS.

**Table 5:** Statistics of the *de novo* transcriptome assembly for each species using Trinity assembler.

	**Species**	***A. gummiferum***	***S. longifolium***	***T.glauca-*****FAR**	***T.glauca*****-BDS**
	**Individual reference**	**Ag3**	**Syl10**	**Tg4**	**V6**
**Counts of transcripts**	Total number of trinity genes (unigene)	84,919	64,716	76,982	53,527
	Total number of trinity transcripts	117,839	89,780	108,823	74,684
	Percent GC	45.86	46.37	44.45	46.21
**Stats based on all transcript contigs**	Contig N50	1,378	1,406	1,396	1,315
	Median contig length (bp)	501	530	547	525
	Average contig length (bp)	843.45	867.27	876.12	839.11
	Total assembled bases	99,391,026	77,863,296	95,341,718	62,667,790
**Stats based on only LONGEST ISOFORM per ׳GENE׳**	Contig N50	1021	1219	1263	1199
	Median contig length (bp)	386	402	421	415
	Average contig length (bp)	672.69	727.09	755.46	734.46
	Total number of assembled bases	57,124,398	47,054,345	58,156,816	39,313,402

#### 2.5.2 Aligning to the *de novo* transcriptomes

We used the Burrows-Wheeler alignment tool (BWA-MEM) to map the cleaned reads from the 24 samples to the corresponding *de novo* transcriptome assemblies of each species [13]. The read aligner, BWA-MEM, aligns each mate of a paired-end read at the same time and produces SAM/BAM files containing the alignments. Samtools view from the SAMtools 1.3 package (http://www.htslib.org/doc/samtools-1.1.html) was used to sort and index the BAM files by coordinate and remove multimapped reads (parameters -q 0 -F 4). Alignment statistics for the mapping to *de novo* transcriptome assembles are displayed in Table 3.

#### 2.5.3 Calling SNPs using *de novo* transcriptome assemblies

The analysis pipeline we used is illustrated in Fig. 4. We processed the aligned reads to a cleaning process with the Galaxy [14] instance of the South Green platform http://galaxy.southgreen.fr/galaxy/. Based upon established GATK best practices, we used Realigner targer creator and IndelRealigner programs (LOD=5) to fix the misalignments due to the mapping process and perform a local realignment near the INDELS [15], [16]. Variant calling was launched both with Unified Genotyper (GATK), using a minimum phred-scaled confidence threshold of 30 [6], and an in-house scripts (Martin et al., *in prep.*, Baurens et al., *in prep.*). We obtained between 600 and 900 thousands of SNPs with Unified Genotyper for each species, and the in-house script identified between 9 and 12 millions of SNPs (Supplementary Table 4).

#### 2.5.4 Variant filtering from mapping to the *de novo* transcriptomes

We applied the same filtering process as explained earlier with the variant filtering from mapping to *E. grandis* genome. We compared the overlap among SNP positions obtained for each calling methods using vcf-compare from VCFtools [10] (Table 6). With *de novo* transcriptomes as reference for mapping, we found around 2/3 of the SNPs identified by Unified Genotyper (GATK) and in-house methods sharing identical positions on *A. gummiferum* and *S. longifolium* (Table 6). The proportion of identical SNPs positions is even bigger with *T. glauca-BDS* and *T.glauca-FAR*, up to 96% using the inhouse-script and 83% using Unified Genotyper (Table 6).

**Table 6:** Overlapped and uniques SNPs called using two different calling methods (GATK and in-house script) from mapping using *de novo* transcriptomes.

Species	Methods	filtered SNP counts	% unique SNP positions	% shared SNP positions between GATK and inhouse script methods
*A. gummiferum*	GATK (Unified Genotyper)	65,623	34	66
	Inhouse script	64,098	33	67
*S. longifolium*	GATK (Unified Genotyper)	84,242	34	66
	Inhouse script	78,612	29	71
*T. glauca*-BDS	GATK (Unified Genotyper)	89,791	38	62
	Inhouse script	57,835	4	96
*T. glauca*-FAR	GATK (Unified Genotyper)	94,274	17	83
	Inhouse script	108,495	27	73

### 2.6 Differential gene expression analysis in infected versus non-infected plants

EdgeR (Bioconductor package) was used to identify Differentially Expressed Genes (DEGs) in the three species aligned with the two types of reference (*E. grandis* genome and *de novo* transcriptome) [17].

From the raw read counts, EdgeR normalized the size of the sample libraries and computed genewise tests for differences in the means between the groups of infected samples versus the group of non-infected samples. It outputted CPM (counts per million), log of fold change (logFC) between the two groups, along with the corresponding p-value and false discovery rate (FDR).

Prior to the normalization step, the genes were filtered. Only the genes whose sum of CPM values (calculated on all the samples) was greater than 1, and which were expressed in at least 2 samples, were kept. Differentially expressed genes were selected based on a fold change ≥ 2 (logFC≥1 and logFC≤1) and an FDR-adjusted *p* value threshold of 0.05. An FDR of 0.05 implied that we were willing to accept that 5% of the differentially expressed genes were false positives.

The total number of identified differentially expressed genes, along with their up-/down-regulation, are summarized in Table 7 and displayed as a plotSmear in Supplementary Figure 1. In EdgeR, dispersion was estimated on a common dispersion basis for all species. The differential expression of genes was analyzed in infected individuals compared to non-infected ones. When using the *E. grandis* reference genome we showed that 12.69% of the total expressed genes were differentially expressed in *A. gummiferum*, 10.32% in *S. longifolium*, 2.45% in *T. glauca-*BDS and 1.75% in T.glauca-*FAR*. When using the *de novo* transcriptome of each species, around 5.6 % of the genes were differentially expressed in *A. gummiferum*, 8.46% in *S. longifolium*, 1.37% in *T. glauca*-BDS and 1.24% in *T.glauca*-FAR.

**Table 7:** Differentially expressed gene resulting from EdgeR.

Reference for reads alignment	Species	Total number of genes	Differentially expressed genes (common dispersion)	over-expressed genes (LogFC≥1)	under-expressed genes (LogFC≤-1)	% of differentially expressed genes
*E. grandis*	*A. gummiferum*	27,294	3463	2792	671	12.69
	*S. longifolium*	26,626	2747	1.768	979	10.32
	*T. glauca*-FAR	23,622	413	234	179	1.75
	*T. galuca*-BDS	27,014	662	609	53	2.45
*de novo* transcriptome	*A. gummiferum*	84,919	4751	2994	1757	5.59
	*S. longifolium*	39,929	3379	2063	1316	8.46
	*T. glauca*-FAR	31,379	388	243	145	1.24
	*T. galuca*-BDS	36,047	493	400	93	1.37

**Fig. 5:**
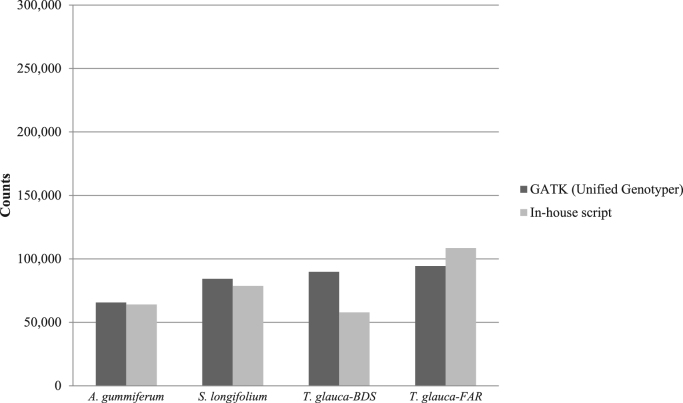
Numbers of SNPs after filtering steps per calling methods and using the *E. grandis* genome as reference for mapping.

**Fig. 6:**
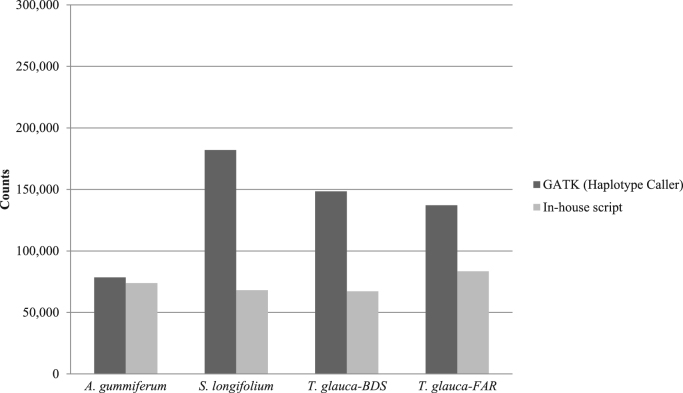
Numbers of SNPs per calling methods and for each studied species using de novo transcriptome of each species as reference for mapping.

As gene expression differences existed between the two groups of individuals (non-infected/infected), it should be expected that biological replicates of the same condition will cluster together. We used a multidimensional scaling (MDS) plot to see a spatial configuration of how similar or dissimilar the non-infected and infected individuals were according to the reference used (Supplementary figure 2). We observed clustering of individuals with the same phenotype for *A. gummiferum* and *T. glauca-FAR* (Supplementary figure 2-A-B and E-F), while for *S. longifolium* the individual Syl10, initially considered non-infected, grouped with infected individuals (Supplementary figure 2-C-D). For *T. glauca*-BDS, the infected individuals had very similar expression patterns, while the non-infected individuals were dispersed (Supplementary figure 2-G-H) (Fig. 5, Fig. 6).

**Fig. 7:**
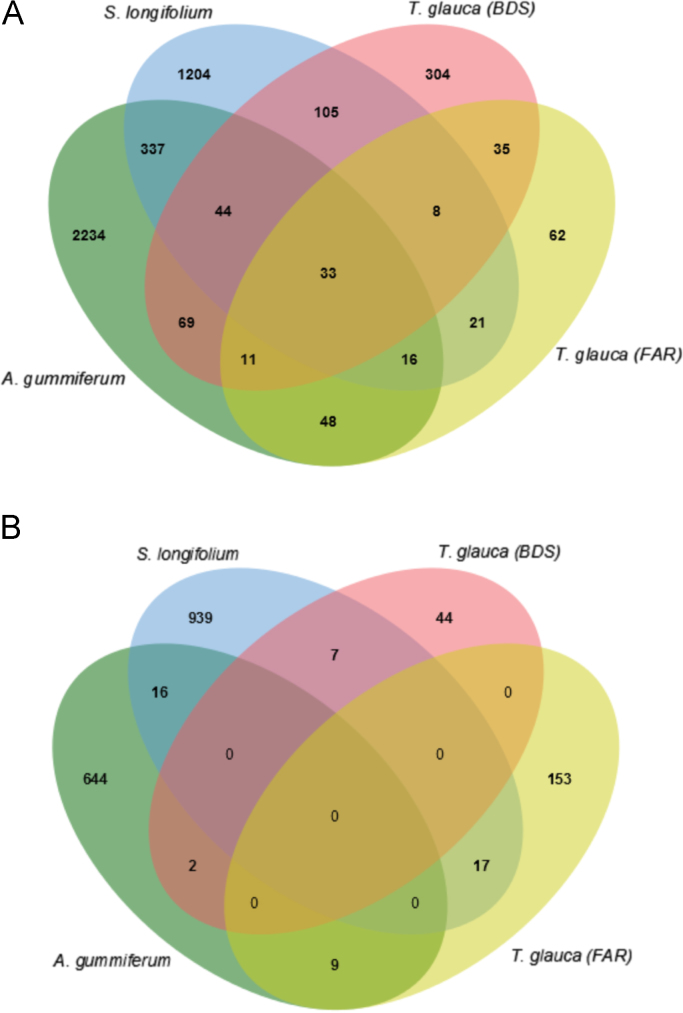
Venn diagram showing the differentially expressed genes in *A. gummiferum*, *S. longifolium* and *T. glauca* (FAR and BDS) using alignments with *E. grandis* reference genome. (A) diagram is for over-expressed genes and (B) diagram for under-expressed ones. Over or under expressed genes means that these genes are differentially expressed for the infected samples.

When using the *E. grandis* genome as a reference for mapping, a cross-comparison of differentially expressed genes (DEGs) was presented as a Venn diagram illustrating the overlapped DEGs (Fig. 7). We found 33 over-expressed genes in infected individuals that overlapped between the three species *A. gummiferum*, *S. longifolium* and *T. glauca* (FAR/BDS) (Table 8), but only 28 had an identified product. No under-expressed genes were found in common between the three species, suggesting that the resistance process toward *A. psidii* is potentially specific to each species. Of the 33 over-expressed genes in infected plants, we found several genes potentially involved in disease response processes (Table 8), such as LOC104438326 coding for a pathogenesis-related protein STH-2-like and various chitinase coding genes (LOC104415213, LOC104419011, LOC104456214, LOC104456215, LOC104456217, LOC104456219, LOC104456220, LOC104456221, LOC104456223). Chitinases are enzymes that hydrolyze the polymer chitin from many chitinolytic biotic aggressors (fungi, bacteria, viruses, viroids) [18]. They are considered as pathogenesis-related proteins playing a crucial role in resistance against pathogens [19]. We also identified gene LOC104445691 coding for a probable WRKY transcription factor 31 isoform X1. This transcription factor specifically interacts with cis-acting elements of plant defense genes that are expressed in reaction to an elicitor. Elicitors are compounds of pathogen origin stimulating any type of plant defense.

**Table 8:** List of common differential expressed genes between *A. gummiferum*, *T. glauca* and *S. longifolium* using *E. grandis* reference genome.

Gene name	Scaffold	Description	Position	
			Begin	End
LOC104415198	scaffold0008	major allergen Pru ar 1-like	57984411	57985238
LOC104415200	scaffold0008	major allergen Pru ar 1-like	57981077	57981765
LOC104415201	scaffold0008	major allergen Pru ar 1-like	57988552	57989434
LOC104415202	scaffold0008	major allergen Pru ar 1-like	58040706	58041532
LOC104415205	scaffold0008	major allergen Pru ar 1-like	58026813	58027698
LOC104415206	scaffold0008	major allergen Pru ar 1-like	58052364	58053248
LOC104415209	scaffold0008	major allergen Pru ar 1-like	58071441	58072223
LOC104415211	scaffold0008	major allergen Pru ar 1-like	58078042	58078973
LOC104415212	scaffold0008	major allergen Pru ar 1-like	58081746	58082621
LOC104415213	scaffold0008	major allergen Pru ar 1-like	58084821	58085687
LOC104419011	scaffold0009	endochitinase-like	25149486	25151347
LOC104422218	scaffold0010	uncharacterized protein	21581164	21582481
LOC104425880	scaffold0011	miraculin-like	30418678	30419730
LOC104428733	scaffold0045	polyphenol oxidase%2C chloroplastic-like	403715	406482
LOC104430480	scaffold0001	cationic peroxidase 1-like	10988229	10991371
LOC104438326	scaffold0001	pathogenesis-related protein STH-2-like	1819744	1820574
LOC104441046	scaffold0004	polyphenol oxidase%2C chloroplastic-like	11894466	11897452
LOC104445691	scaffold0005	probable WRKY transcription factor 31 isoform X1	69013730	69016591
LOC104447583	scaffold0001	1-aminocyclopropane-1-carboxylate oxidase homolog 4-like	37081804	37083288
LOC104447594	scaffold0001	1-aminocyclopropane-1-carboxylate oxidase homolog 4-like	37094712	37096232
LOC104450568	scaffold0001	lichenase	4937091	4938832
LOC104456214	scaffold0008	endochitinase PR4-like	4453707	4454827
LOC104456215	scaffold0008	chitinase 6-like	4487298	4488395
LOC104456217	scaffold0008	endochitinase PR4-like	4496392	4497507
LOC104456219	scaffold0008	endochitinase PR4-like	4522275	4523440
LOC104456220	scaffold0008	endochitinase PR4-like	4533169	4534322
LOC104456221	scaffold0008	chitinase 6-like	4546083	4547171
LOC104456223	scaffold0008	endochitinase PR4-like	4553763	4554816

### 2.7 Annotation

We ran FrameDP V1.2.2 software with default parameters [20], for the prediction of coding regions in the unigenes using the *E. grandis* protein database from Universal Protein Resource (UniProt-Swissprot). FrameDP V1.2.2 software automatically reverse-complement the sequences, however all the previous analysis were performed on the initial DNA strand orientation. Therefore, we changed the reverse-complemented orientation from FrameDP results to the initial genes orientation. The polypeptides sequences obtained were submitted to the Basic Local Alignment Search Tool (BLAST) to search against homologs in the UnitProt (Swiss-Prot and Trembl) databases. We identified 45,517 protein-coding genes in *T. glauca*-BDS, 50,173 protein-coding genes in *S. longifolium*, 57,366 protein-coding genes in *T. glauca*-FAR and 65,410 protein-coding gene in *A. gummiferum*.

### 2.8 Genome browser

**Fig. 8:**
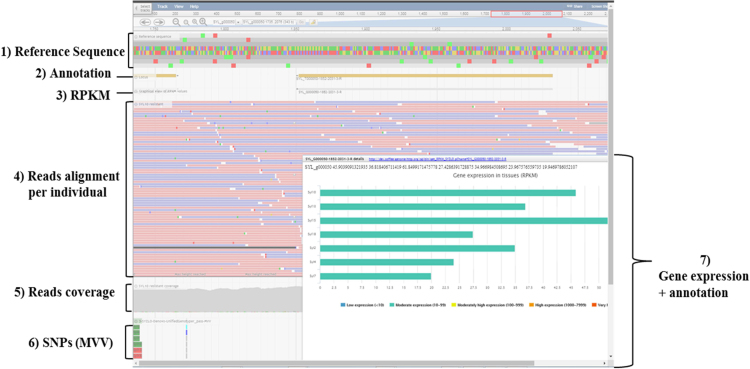
Screenshot of the JBrowse of *Syzygium longiflorum*.

To make easier the analysis of the massive amounts of genetic information that was generated during this study, we used the JBrowse tool from GMOD project (Generic Model Organism Database project) [21]. JBrowse is a web-based genome browser, allowing interactively visualizing and exploring a large genomic dataset [22]. Each track consists of a particular type of sequence feature along a reference sequence, as showed in Fig. 8. We deployed five different JBrowses with the data from each species, taking first the *E. grandis* genome as a reference for alignment, then the *de novo* transcriptomes (*A. gummiferum*, *S. longifolium*, *T. glauca*-FAR and *T. glauca*-BDS). To build the JBrowses, we rely on a released workflow [23] and we imported the following files: reference sequence files (*de novo* transcriptomes (.faa) or *E. grandis* reference genome (.fna), alignments files (.bam), variants calling files (.vcf), annotations files (.gff3), and EdgeR output files containing the RPKM (expression level) of each locus for the samples (.tsv). To load the following data we used the following perl scripts provided by the JBrowse developers: prepare-refseqs.pl for fasta files and flatfile-to-json.pl for .gff3 and .tsv files. To load the others data types we had to edit two files tracks.conf and trackList.json, respectively displaying the dataset-specific names and configuration. We also used a JBrowse plugin called MultiVariantViewer (multivariantviewerjbrowsepluging) to display the corresponding genotype for each SNP from each individual of this study (https://github.com/elsiklab/multivariantviewer). These genotypes are displayed in three different colors: cyan for heterozygotes, grey for homozygotes for the reference allele and deep blue for the homozygotes of the alternative allele. To load the MVV tracks (sample name, category, and genotype), we edited the trackList.json from each JBrowse. The JBrowses were integrated into the open source CMS Drupal [24], which is distributed under the terms of the GNE’s Not Unix General Public License (http://myrtaceae-omics.southgreen.fr).
