# Supplementary material for: Transcriptome data from three endemic Myrtaceae species from New Caledonia displaying contrasting responses to myrtle rust (Austropuccinia psidii)
Source: Data Brief. 2019 Jan 3;22:794–811. doi: 10.1016/j.dib.2018.12.080 (PMC6362868; doi:10.1016/j.dib.2018.12.080)
Supplement: Supplementary file 2 — Supplementary material [file mmc2.pdf]

Supplementary figure 1: EdgeR's plotSmear function plots the log-fold change (i.e., the log ratio of normalized expression levels between non-infected and infected) against the log counts per million (CPM).

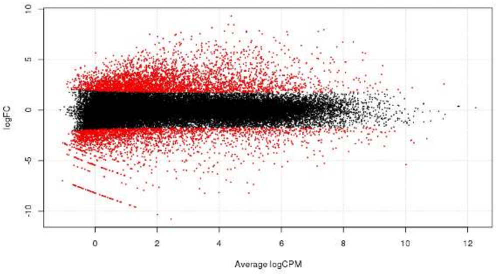

A. PlotSmear from A. gummiferum using the de novo transcriptome assembly as reference for alignment.

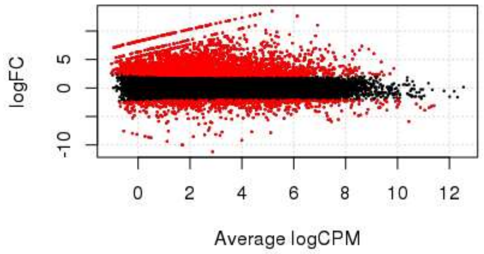

B. PlotSmear from A. gummiferum using the E. grandis genome as reference for alignment.

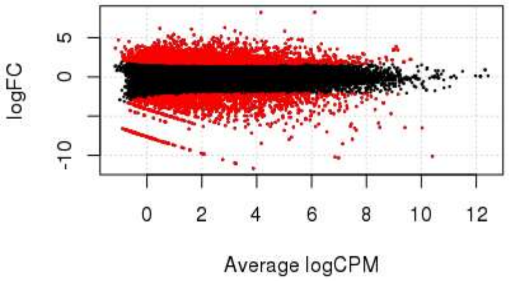

C. PlotSmear from S. longifolium using the de novo transcriptome assembly for alignment.

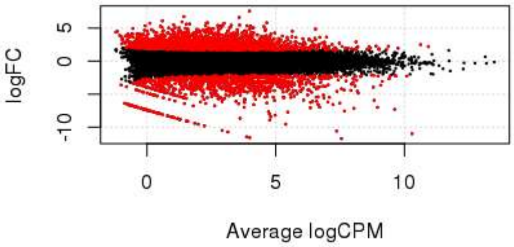

D. PlotSmear from S. longifolium using the E. grandis genome as reference for alignment.

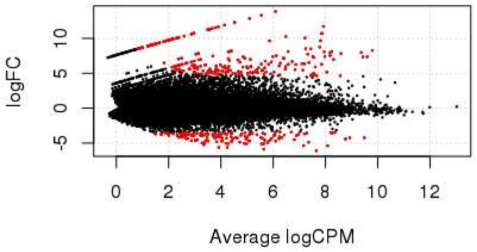

E. PlotSmear from T. glauca-FAR using the de novo transcriptome assembly for alignment.

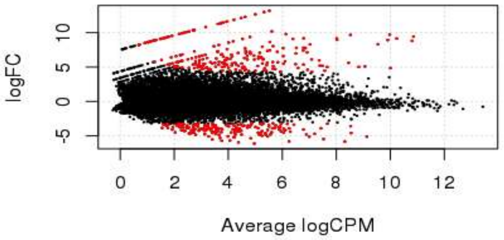

F. PlotSmear from T. glauca-FAR using the E. grandis genome as reference for alignment.

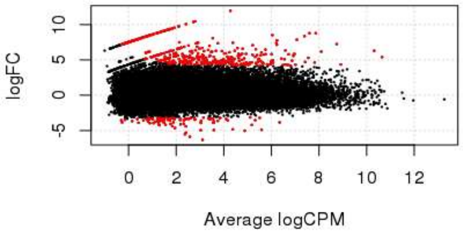

G. PlotSmear from T. glauca-BDS using the de novo transcriptome assembly for alignment.

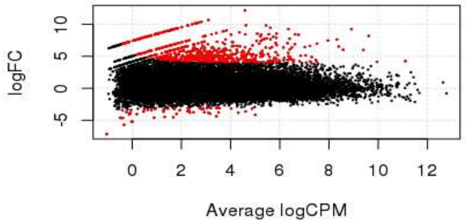

H. PlotSmear from T. glauca-BDS using the E. grandis genome as reference for alignment.
