# Supplementary material for: Transcriptome data from three endemic Myrtaceae species from New Caledonia displaying contrasting responses to myrtle rust (Austropuccinia psidii)
Source: Data Brief. 2019 Jan 3;22:794–811. doi: 10.1016/j.dib.2018.12.080 (PMC6362868; doi:10.1016/j.dib.2018.12.080)
Supplement: Supplementary file 3 — Supplementary material [file mmc3.pdf]

Supplementary figure 2: Plots of samples relations by using multidimensional scaling plot (MDS) from EdgeR. Infected individuals are in red, and non-infected individuals are in black.

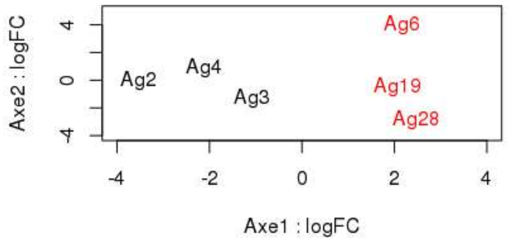

A. MDS plot from *A. gummiferum* using the de novo transcriptome assembly as reference for alignment.

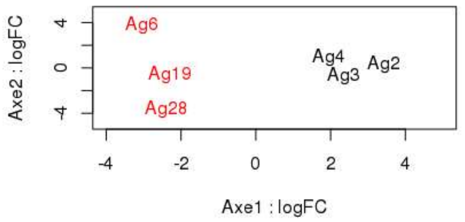

B. MDS plot from *A. gummiferum* using the E. grandis genome as reference for alignment.

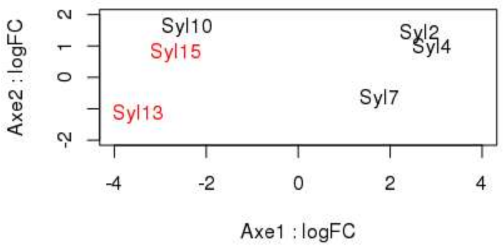

C. MDS plot from *S. longifolium* using the de novo transcriptome assembly for alignment.

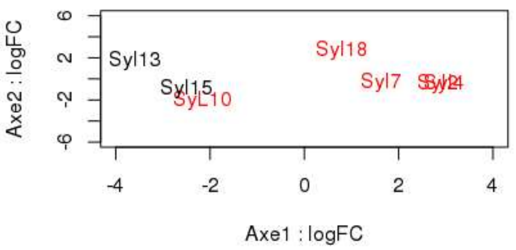

D. MDS plot from *S. longifolium* using the E. grandis genome as reference for alignment.

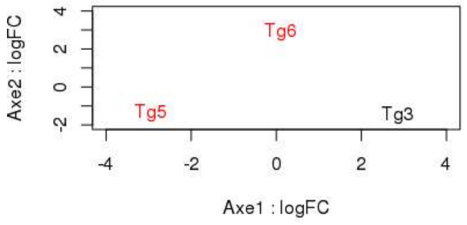

E. MDS plot from *T. glauca*-FAR using the de novo transcriptome assembly for alignment.

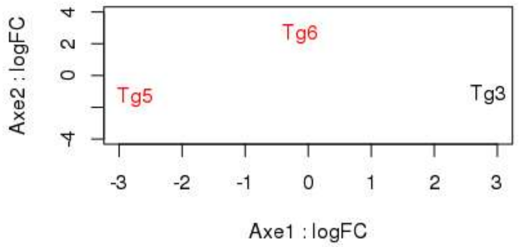

F. MDS plot from *T. glauca*-FAR using the E. grandis genome as reference for alignment.

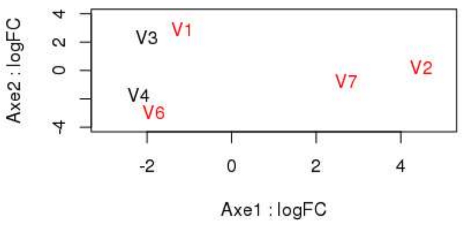

G. MDS plot from *T. glauca*-BDS using the de novo transcriptome assembly for alignment.

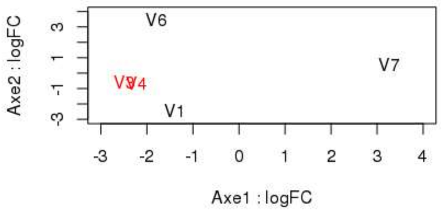

H. MDS plot from *T. glauca*-BDS using the E. grandis genome as reference for alignment.
