# Supplementary material for: Transcriptome data from three endemic Myrtaceae species from New Caledonia displaying contrasting responses to myrtle rust (Austropuccinia psidii)
Source: Data Brief. 2019 Jan 3;22:794–811. doi: 10.1016/j.dib.2018.12.080 (PMC6362868; doi:10.1016/j.dib.2018.12.080)
Supplement: Supplementary file 4 — Supplementary material [file mmc4.docx]

Supplementary Table 1: Detailed of RNA extracts

| **Sample name** | **ID sampling** | **Biological material** | **Organism** | **BioAnalyzeur RNA concentration (ng/µl)** | **rRNA Ratio [25s/18s]** | **RIN** |
| --- | --- | --- | --- | --- | --- | --- |
| Sample 1 | Ag19 | leaf | *Arillastrum gummiferum* | 281 | 3 | 8,2 |
| Sample 2 | Ag28 | leaf | *Arillastrum gummiferum* | 98 | 3,4 | 8,2 |
| Sample 3 | Ag2 | leaf | *Arillastrum gummiferum* | 353 | 2,3 | 6,9 |
| Sample 4 | Ag3 | leaf | *Arillastrum gummiferum* | 473 | 3,1 | 7,4 |
| Sample 5 | Ag4 | leaf | *Arillastrum gummiferum* | 531 | 2,9 | 7,4 |
| Sample 6 | Ag6 | leaf | *Arillastrum gummiferum* | 843 | 1,5 | 7,8 |
| Sample 7 | Syl10 | leaf | *Syzygium longifolium* | 414 | 2,3 | 7,7 |
| Sample 8 | Syl13 | leaf | *Syzygium longifolium* | 1248 | 3,4 | 7,9 |
| Sample 9 | Syl15 | leaf | *Syzygium longifolium* | 499 | 4,3 | 8 |
| Sample 10 | Syl18 | leaf | *Syzygium longifolium* | 602 | 1,8 | 7,5 |
| Sample 11 | Syl2 | leaf | *Syzygium longifolium* | 790 | 2,1 | 8,2 |
| Sample 12 | Syl4 | leaf | *Syzygium longifolium* | 667 | 1,8 | 8 |
| Sample 13 | Syl7 | leaf | *Syzygium longifolium* | 760 | 2,8 | 7,7 |
| Sample 14 | Tg2 | leaf | *Tristaniopsis glauca* (FAR) | 215 | 3,6 | 7,5 |
| Sample 15 | Tg3 | leaf | *Tristaniopsis glauca* (FAR) | 197 | 2,6 | 7,4 |
| Sample 16 | Tg4 | leaf | *Tristaniopsis glauca* (FAR) | 117 | 3,2 | 8,1 |
| Sample 17 | Tg5 | leaf | *Tristaniopsis glauca* (FAR) | 249 | 2,9 | 7,8 |
| Sample 18 | Tg6 | leaf | *Tristaniopsis glauca* (FAR) | 309 | 3,3 | 8 |
| Sample 19 | V1 | leaf | *Tristaniopsis glauca* (BDS) | 114 | 3,4 | 8,4 |
| Sample 20 | V2 | leaf | *Tristaniopsis glauca* (BDS) | 135 | 3,2 | 7,9 |
| Sample 21 | V3 | leaf | *Tristaniopsis glauca* (BDS) | 249 | 2,9 | 8,3 |
| Sample 22 | V4 | leaf | *Tristaniopsis glauca* (BDS) | 236 | 3,9 | 8,2 |
| Sample 23 | V6 | leaf | *Tristaniopsis glauca* (BDS) | 347 | 2,3 | 8,3 |
| Sample 24 | V7 | leaf | *Tristaniopsis glauca* (BDS) | 108 | 1,8 | 7,9 |

Supplementary table 2: Number of raw and cleaned reads. The individual used for *de novo* transcriptome assembly are shown in yellow.

|  |  |  | Rawreads | | Cutadapt | | Quality filter | | Synchronized paired | | |
| --- | --- | --- | --- | --- | --- | --- | --- | --- | --- | --- | --- |
| Species | libraries | pair | Number of read | Total reads | Number of read | Total reads | Number of read | Total reads | Number of read in paired sequences | Number of read in single sequence | Total reads |
| *A.,gummiferum* | Ag19 | R1.fastq.gz | 13,999,356 | 176,074,893 | 13,979,390 | 176,550,782 | 13,826,858 | 172,686,969 | 13,487,790 | 420,309 | 172,686,969 |
|  |  | R2.fastq.gz | 13,999,356 |  | 13,963,517 |  | 13,569,031 |  | 13,487,790 |  |  |
|  | Ag28 | R1.fastq.gz | 15,117,850 |  | 15,106,309 |  | 14,952,929 |  | 14,614,463 | 424,045 |  |
|  |  | R2.fastq.gz | 15,117,850 |  | 15,090,520 |  | 14,700,042 |  | 14,614,463 |  |  |
|  | Ag2 | R1.fastq.gz | 16,737,435 |  | 16,720,235 |  | 16,077,560 |  | 15,717,141 | 756,610 |  |
|  |  | R2.fastq.gz | 16,737,435 |  | 16,703,178 |  | 16,113,332 |  | 15,717,141 |  |  |
|  | Ag3 | R1.fastq.gz | 17,559,520 |  | 17,536,161 |  | 17,368,035 |  | 16,974,642 | 485,658 |  |
|  |  | R2.fastq.gz | 16,737,435 |  | 17,518,848 |  | 17,066,907 |  | 16,974,642 |  |  |
|  | Ag4 | R1.fastq.gz | 11,841,969 |  | 11,832,007 |  | 11,715,154 |  | 11,447,310 | 330,994 |  |
|  |  | R2.fastq.gz | 11,841,969 |  | 11,823,208 |  | 11,510,460 |  | 11,447,310 |  |  |
|  | Ag6 | R1.fastq.gz | 13,192,359 |  | 13,146,912 |  | 13,001,753 |  | 12,704,802 | 377,057 |  |
|  |  | R2.fastq.gz | 13,192,359 |  | 13,130,497 |  | 12,784,908 |  | 12,704,802 |  |  |
| *S.,longifolium* | Syl10 | R1.fastq.gz | 18,410,200 | 200,293,564 | 18,399,611 | 199,924,373 | 18,214,298 | 196,307,059 | 17,786,867 | 531,480 | 196,307,059 |
|  |  | R2.fastq.gz | 18,410,200 |  | 18,380,383 |  | 17,890,916 |  | 17,786,867 |  |  |
|  | Syl13 | R1.fastq.gz | 11,906,944 |  | 11,892,642 |  | 11,784,325 |  | 11,584,279 | 267,325 |  |
|  |  | R2.fastq.gz | 11,906,944 |  | 11,880,199 |  | 11,651,558 |  | 11,584,279 |  |  |
|  | Syl15 | R1.fastq.gz | 12,185,308 |  | 12,169,050 |  | 12,038,801 |  | 11,790,027 | 321,993 |  |
|  |  | R2.fastq.gz | 12,185,308 |  | 12,155,770 |  | 11,863,246 |  | 11,790,027 |  |  |
|  | Syl18 | R1.fastq.gz | 13,361,746 |  | 13,348,180 |  | 13,210,653 |  | 12,827,489 | 448,322 |  |
|  |  | R2.fastq.gz | 13,361,746 |  | 13,328,387 |  | 12,892,647 |  | 12,827,489 |  |  |
|  | Syl2 | R1.fastq.gz | 15,528,002 |  | 15,511,653 |  | 15,357,301 |  | 15,028,873 | 417,797 |  |
|  |  | R2.fastq.gz | 15,528,002 |  | 15,496,078 |  | 15,118,242 |  | 15,028,873 |  |  |
|  | Syl4 | R1.fastq.gz | 15,452,459 |  | 15,412,531 |  | 15,242,629 |  | 14,834,105 | 497,267 |  |
|  |  | R2.fastq.gz | 15,452,459 |  | 15,391,995 |  | 14,922,848 |  | 14,834,105 |  |  |
|  | Syl7 | R1.fastq.gz | 13,302,123 |  | 13,286,296 |  | 13,152,135 |  | 12,889,198 | 341,199 |  |
|  |  | R2.fastq.gz | 13,302,123 |  | 13,271,598 |  | 12,967,460 |  | 12,889,198 |  |  |

Supplementary table 2 continued from previous page

|  |  |  | Rawreads | | Cutadapt | | Quality filter | | Synchronized paired | | |
| --- | --- | --- | --- | --- | --- | --- | --- | --- | --- | --- | --- |
| Species | libraries | pair | Number of read | Total reads | Number of read | Total reads | Number of read | Total reads | Number of read in paired sequences | Number of read in single sequence | Total reads |
| *T.glauca-*FAR | Tg2 | R1.fastq.gz | 12,628,209 | 137,602,172 | 12,619,729 | 137,325,570 | 12,506,099 | 134,446,070 | 12,256,035 | 317,804 | 134,446,070 |
|  |  | R2.fastq.gz | 12,628,209 |  | 12,607,318 |  | 12,323,775 |  | 12,256,035 |  |  |
|  | Tg3 | R1.fastq.gz | 14,403,325 |  | 14,391,609 |  | 14,257,034 |  | 13,943,792 | 386,364 |  |
|  |  | R2.fastq.gz | 14,403,325 |  | 14,380,994 |  | 14,016,914 |  | 13,943,792 |  |  |
|  | Tg4 | R1.fastq.gz | 16,207,008 |  | 16,174,551 |  | 15,618,141 |  | 15,124,929 | 784,505 |  |
|  |  | R2.fastq.gz | 16,207,008 |  | 16,135,866 |  | 15,416,222 |  | 15,124,929 |  |  |
|  | Tg5 | R1.fastq.gz | 13,874,232 |  | 13,841,109 |  | 13,716,610 |  | 13,528,269 | 267,298 |  |
|  |  | R2.fastq.gz | 13,874,232 |  | 13,830,270 |  | 13,607,226 |  | 13,528,269 |  |  |
|  | Tg6 | R1.fastq.gz | 11,688,312 |  | 11,676,931 |  | 11,570,381 |  | 11,352,254 | 279,541 |  |
|  |  | R2.fastq.gz | 11,688,312 |  | 11,667,193 |  | 11,413,668 |  | 11,352,254 |  |  |
| *T.glauca*-BDS | V1 | R1.fastq.gz | 8,858,469 | 154,194,966 | 8,817,505 | 153,676,220 | 8,741,346 | 150,931,462 | 8,643,046 | 147,634 | 150,931,462 |
|  |  | R2.fastq.gz | 8,858,469 |  | 8,807,090 |  | 8,692,380 |  | 8,643,046 |  |  |
|  | V2 | R1.fastq.gz | 13,962,685 |  | 13,942,810 |  | 13,817,346 |  | 13,589,262 | 301,974 |  |
|  |  | R2.fastq.gz | 13,962,685 |  | 13,931,957 |  | 13,663,152 |  | 13,589,262 |  |  |
|  | V3 | R1.fastq.gz | 12,086,290 |  | 12,073,243 |  | 11,960,434 |  | 11,772,532 | 256,341 |  |
|  |  | R2.fastq.gz | 12,086,290 |  | 12,062,913 |  | 11,840,971 |  | 11,772,532 |  |  |
|  | V4 | R1.fastq.gz | 11,998,827 |  | 11,936,661 |  | 11,528,882 |  | 11,209,706 | 526,788 |  |
|  |  | R2.fastq.gz | 11,998,827 |  | 11,870,792 |  | 11,417,318 |  | 11,209,706 |  |  |
|  | V6 | R1.fastq.gz | 15,648,452 |  | 15,626,992 |  | 15,480,924 |  | 15,213,148 | 356,039 |  |
|  |  | R2.fastq.gz | 15,648,452 |  | 15,609,495 |  | 15,301,411 |  | 15,213,148 |  |  |
|  | V7 | R1.fastq.gz | 14,542,760 |  | 14,511,762 |  | 14,367,592 |  | 14,033,652 | 419,994 |  |
|  |  | R2.fastq.gz | 14,542,760 |  | 14,485,000 |  | 14,119,706 |  | 14,033,652 |  |  |

Supplementary Table 3: Number of SNP following each filtering step and using *E. grandis* genome as reference for mapping.

| Reference type | SNP calling method | Species | raw | prefilter | VCFfilter | Monomorphfilter | GATKfilter |
| --- | --- | --- | --- | --- | --- | --- | --- |
| *E.grandis* genome | GATK(HaplotypeCaller) | *A.gummiferum* | 5,641,707 |  | 411,927 | 157,905 | 78,468 |
|  |  | *S.longifolium* | 2,550,459 |  | 342,695 | 270,689 | 181,967 |
|  |  | *T.glauca*-BDS | 3,076,802 |  | 280,610 | 220,237 | 148,484 |
|  |  | *T.glauca*-FAR | 3,076,802 |  | 382,358 | 157,262 | 137,073 |
|  | Inhousescript | *A.gummiferum* | 10,768,345 | 2,627,198 | 825,906 | 90,879 | 73,765 |
|  |  | *S.longifolium* | 8,860,083 | 2,246,428 | 723,591 | 79,486 | 68,106 |
|  |  | *T.glauca*-BDS | 11,671,338 | 2,866,631 | 708,324 | 78,468 | 67,115 |
|  |  | *T.glauca*-FAR | 11,671,338 | 2,866,631 | 877,238 | 100,575 | 83,423 |

Supplementary Table 4: Number of SNP following each filtering step and using *de novo* transcriptome as reference for mapping.

| Reference type | SNP calling method | Species | raw | prefilter | VCFfilter | Monomorphfilter | GATKfilter |
| --- | --- | --- | --- | --- | --- | --- | --- |
| *de novo* transcriptome | GATK(UnifiedGenotyper) | *A.gummiferum* | 648,301 |  | 199,892 | 95,741 | 65,623 |
|  |  | *S.longifolium* | 602,223 |  | 192,069 | 111,489 | 84,242 |
|  |  | *T.glauca*-BDS | 935,693 |  | 257,152 | 122,951 | 89,791 |
|  |  | *T.glauca*-FAR | 783,330 |  | 252,744 | 129,592 | 94,274 |
|  | Inhousescript | *A.gummiferum* | 10,501,327 | 2,140,954 | 1,451,164 | 82,280 | 64,098 |
|  |  | *S.longifolium* | 10,210,671 | 2,141,428 | 1,514,849 | 91,416 | 78,612 |
|  |  | *T.glauca*-BDS | 9,436,592 | 2,100,408 | 1,409,764 | 68,608 | 57,835 |
|  |  | *T.glauca*-FAR | 12,433,821 | 2,643,534 | 1,650,084 | 133,857 | 108,495 |
